# The Association of Plasma Osmolarity with No-Reflow in Patients with ST Elevation Myocardial Infarction: A Retrospective Cohort Study

**DOI:** 10.5152/eurasianjmed.2024.23143

**Published:** 2024-02-01

**Authors:** Şerif Hamideyin, İnanç Artaç

**Affiliations:** Department of Cardiology, Kafkas University Faculty of Medicine, Kars, Turkey

**Keywords:** No-reflow phenomenon, plasma osmolarity, percutaneous coronary intervention, ST elevation myocardial infarction

## Abstract

**Background:**

In this study, we sought to examine the statistical association of plasma osmolarity with no-reflow development in patients with ST-segment elevation myocardial infarction (STEMI) who were treated with primary percutaneous coronary intervention (pPCI).

**Methods:**

In this retrospective study, we included data from 1294 consecutive STEMI patients who have undergone pPCI. For each patient, we measured the plasma osmolarity using the following equation: 2 × sodium + 0.9 glucose + 0.93 × urea × 0.5.

**Results:**

Occurrence of angiographic no-reflow was 21.7% (n = 281) in the study. The mean plasma osmolarity level was significantly higher in patients with no-reflow compared to those without no-reflow (300.6 ± 9.4 mOsmol/L versus 292.8 ± 10.5 mOsmol/L, *P* < .001, respectively). In multivariate logistic regression analysis, plasma osmolarity was found to be independently related to no-reflow development (odds ratio: 1.061; 95% CI, 1.045-1.076; *P *< .001). According to the receiver operating characteristic curve analysis, a plasma osmolarity level greater than 290.2 mOsmol/L was identified as the optimal value for predicting the occurrence of no-reflow. This cutoff demonstrated a sensitivity of 91.8% and a specificity of 45.8%.

**Conclusion:**

This is the first study to establish an independent relationship between higher plasma osmolarity and the development of no-reflow in patients with STEMI who have undergone pPCI. This finding suggests that plasma osmolarity may be a useful marker for the prediction of no-reflow in STEMI patients who have undergone pPCI.

**Main Points**

- We examined the relationship between no-reflow and plasma osmolarity, which is easy to calculate.
- We observed that the increase in plasma osmolarity in ST-elevation myocardial infarction patients is associated with an increased incidence of no-reflow and mortality.
- According to our study, high plasma osmolarity can predict no-reflow with a sensitivity of 92% and a specificity of 46%.

## Introduction

In current practice, primary percutaneous coronary intervention (pPCI) is the recommended method of treatment in patients who present with ST-segment elevation myocardial infarction (STEMI), aimed at restoring the thrombolysis in myocardial infarction (TIMI) flow of grade 3 after coronary intervention. However, the reopening of the infarct-related artery (IRA) always does not translate into sufficient myocardial reperfusion in spite of the elimination of the target vessel obstruction. This phenomenon is referred to as no-reflow.^1^ Even if the underlying mechanism of no-reflow is not well understood, and is somewhat complex, transmyocardial damage before pPCI and the size of the related area are major determinants in the development of no-reflow.^2,3^ In addition, there was strong evidence showing that inflammation, elevation of some cytokines and mediators, microvascular obstruction, oxidative stress, reperfusion injury, and the absence of ischemic preconditioning might contribute to the occurrence of such a phenomenon.^2-5^ Moreover, some studies revealed that some clinical risk factors, including older age and increased heart rate, and some angiographic findings, such as the presence of high thrombus burden and the lack of coronary blood flow before pPCI, were independent predictors of no-reflow.^6,7^

Plasma osmolarity has a vital role in the intracellular and extracellular water distribution, and it chiefly hinges on the concentrations of plasma sodium, blood urea nitrogen (BUN), and glucose. Some human and animal in vitro studies have consistently demonstrated that hyperosmolarity, together with arginine vasopressin and copeptin secretion, increases proinflammatory cytokines’ secretion from macrophages (such as interleukin-6 and tumor necrosis factor-*α*), amplifies neutrophil function, increases leukotriene production, and causes endothelial dysfunction and reperfusion injury in the coronary microvasculature.^8-11^ Also, hyperosmolarity due to the increase in plasma glucose and BUN levels could lead to microvascular dysfunction, aggravate platelet-dependent thrombosis, and induce a hypercoagulable condition in the acute setting of myocardial infarction.^12-14^ Taking into consideration these data, we contemplated that there might be a relationship between higher plasma osmolarity and no-reflow development in STEMI patients since all of these mechanisms might have a crucial impact in the pathogenesis of no-reflow. Hence, the purpose of our study was to examine the relationship between plasma osmolarity and no-reflow development in patients with STEMI who were managed with pPCI.

## Material and Methods

### Study Population

A total of 1452 patients with STEMI who had undergone pPCI between December 2019 and March 2022 were retrospectively screened. The patients who had one or more factors such as pregnancy, having been treated with thrombolytic drugs, having had a recent cranial surgery and trauma, active infection(s), prior cerebrovascular accidents, malignancy, severe electrolyte imbalance, autoimmune disease, heart failure, end-stage renal failure, and having undergone emergency coronary aorta by-pass grafting were removed from the study. Additionally, patients whose plasma sodium, glucose, and BUN quantitative measurements were not performed within 8 hours after admittance were also removed. After evaluation concerning the exclusion criteria, a total of 1294 patients, in whom epicardial artery patency was achieved with pPCI, constituted the study’s population. Patients’ laboratory characteristics of demographic and angiographic data were obtained from the medical system database of the hospital. Every single patient was treated as per the current guidelines during the study period. The types of myocardial infarctions were not documented because posterior and right ECGs (electrocardiogram) were not routinely recorded in patients with STEMI. In accordance with the Declaration of Helsinki, the study protocol was implemented and this study was approved by Ethics committee of Kars Kafkas University (Approval No: 80576354-050-99/170, Date: 23/09/2022). While including patients in our retrospectively designed study, we reviewed their medical records and ensured that informed consent for the use of patient data in scientific research had been obtained.

### Laboratory Analysis and Transthoracic Echocardiography

The blood specimens, including plasma sodium, glucose, and BUN, were measured in all patients within 8 hours after admittance and analyzed by utilizing standard biochemical quantitative techniques, with the Beckman Coulter LH 780 Analyzer (Beckman Coulter Ireland Inc., Mervue, Galway, Ireland). The plasma osmolarity was measured using the following equation, which showed mean differences of <1 mOsmol/L compared to the direct measurement of the plasma osmolarity: 2 × sodium + 0.9 glucose + 0.93 × urea × 0.5.^15^

Additionally, an echocardiographic evaluation of the left ventricular (LV) systolic function (ejection fraction) was performed within 24 hours after admission using a Vivid 7 imaging system (GE Vingmed Ultrasound AS, Horten, Norway) to investigate the patients. We utilized the Simpson’s rule to calculate the left ventricular ejection fraction (LVEF).

### Coronary Angiogram and Percutaneous Coronary Intervention

A coronary angiography through the radial or femoral approach was performed in all patients by using the standard 6-French Judkins diagnostic catheters. All patients without contraindications were given an oral loading dose of 8 × 75 mg clopidogrel and 3 × 100 mg acetylsalicylic acid before a coronary angiogram. During coronary intervention, a standard intravenous bolus of high-molecular-weight heparin (HMWH) (70-100 U/kg) was administered with supplementary doses, if required, to ensure that the activated coagulation time was >250 seconds. Although it is not recommended to switch between low-molecular-weight heparin (LMWH) and HMWH, patients preloaded with LMWH were administered the lowest dose within the HMWH range due to challenges in accessing and utilizing the drug. A non-ionic, low-osmolarity contrast medium was used in all procedures. To enable quantitative analysis, all coronary angiograms were digitally recorded and saved in DICOM (Digital Imaging and Communications in Medicine) format (DICOM-viewer; MedCom GmbH, Darmstadt, Germany). Immediately after coronary angiography, all patients underwent revascularization of the IRA with a drug-eluting or bare-metal stent. The management of no-reflow, which can occur during the coronary intervention procedure and is typically treated with adenosine, calcium channel blockers, sodium nitroprusside, glycoprotein IIb/IIIa inhibitors, nitroglycerin, and epinephrine, was determined by the operator’s discretion in alignment with the institutional protocol. Patients who encountered no-reflow during the coronary procedure and exhibited improved blood flow in the final images were classified within the group of patients without no-reflow. Two experienced interventional cardiologists, blinded to all patients’ clinical data, analyzed the TIMI flow grade and myocardial blush grade (MBG) prior to and following the intervention.^16^ If there was any disagreement, the 2 cardiologists examined the coronary angiograms together and reached a consensus. Myocardial blush was graded as described in the studies of Van’t Hof et al.^17^

According to Gibson’s study, thrombus burden was assessed on a scale ranging from the absence of a thrombus (grade 0) to the presence of a significantly large thrombus (grade 5), resulting in arterial obstruction.^18^ In the case of a grade 5 thrombus, the thrombus burden was reclassified from grade 0 to grade 4 after recanalization with a small balloon or a guide wire. The angiographic no-reflow, characterized as a TIMI flow grade of < 3 or 3 with an MBG of 0 to 1, is used in the present study.^19^ Additionally, all patients were medically evaluated in line with the Killip class examination findings.

### Definitions

ST-segment elevation myocardial infarction was defined according to current guidelines.^20^ Hypertensive disease was described as patients receiving antihypertensive therapy or as defined in current hypertension guidelines.^21^Compliance with the diagnostic criteria of the American Diabetes Association guidelines during hospital follow-ups or use of insulin or oral antidiabetic drugs were accepted as diabetes mellitus (DM).^22^ Calculation of the estimated glomerular filtration rate (eGFR) was performed utilizing the equation derived from the Modification of Diet in Renal Diseases study.^23^

### Statistical Analyses

Statistical Package for Social Science Statistics software, version 21.0 for Windows (IBM SPSS Corp.; Armonk, NY, USA), and MedCalc trial version 16.8.4 were utilized to carry out the statistical data analysis. The mean ± SD, or median (25%-75%) value, was used to convey the data. The continuous variable analysis was completed by either the variance analysis (Kruskal–Wallis) or a basic independent *t*-test (Mann*–*Whitney *U*-test). The categorical variable relation was analyzed by the chi-square test. Additionally, the study’s population was categorized into 3 tertiles in line with plasma osmolarity levels. The analysis of variance test was utilized to compare the differences between the groups. All relevant variables were included in a univariate analysis. The independent predictors of no-reflow were found utilizing a multivariate analysis that was a stepwise backward conditional logistic regression analysis. The multicollinearity between plasma osmolarity and its components (blood glucose, BUN, and sodium) was assessed by the eigenvalue and the condition index. No multicollinearity was found between the plasma osmolarity and blood glucose (eigenvalue: 0.100; condition index: 4.35) and BUN levels (eigenvalue: 0.060; condition index: 5.67). However, multicollinearity was found between the plasma osmolarity and the sodium (eigenvalue: < 0.001; condition index: 65.9). Thus, sodium was not incorporated into the multivariate analysis. The power value (1 – *β*) and effect size (Cohen’s *d*) for the study regarding plasma osmolarity were computed utilizing the G*Power software. The power value and effect size were 0.96 and 0.76, respectively. For the purpose of identifying the optimal cutoff level of plasma osmolality for predicting no-reflow, an analysis of the receiver operator curve characteristics was conducted utilizing Youden’s *J* statistic.^24^ The Kaplan–Meier analysis was utilized to generate event-free survival curves, and the plasma osmolarity was differentiated using the log-rank test. The notable result of a two-tailed *P *< .05 was recorded.

## Results

**Figure 1.**
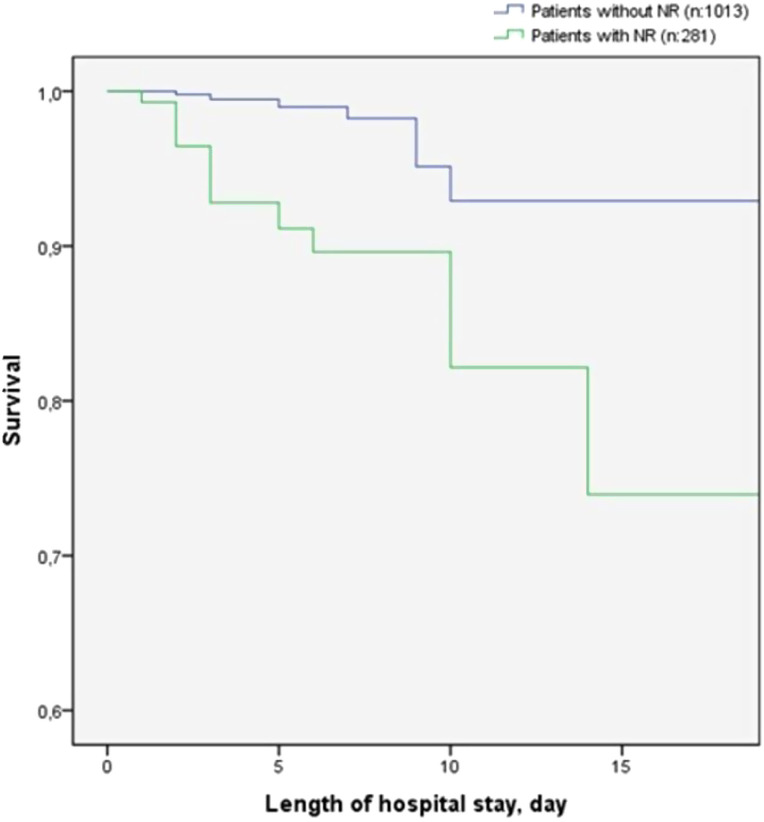
Kaplan–Meier survival analysis for inhospital mortality in patients with no-reflow and without no-reflow.

The average age of 56 ± 12 years was observed in the study population, with 241 of the 1294 patients being female. After pPCI, the development of angiographic no-reflow was 21.7% (n = 281) in the present study. The laboratory findings, baseline demographic characteristics, and echocardiographic and angiographic data of 1294 patients are depicted in Table 1. The patients with no-reflow were older compared to those without no-reflow (*P *< .001). While statistically significant differences were observed for DM and smoking between the groups (*P *< .05 for each), the frequency of hypertension and female gender revealed similar differences (*P *> .05 each). No-reflow patients had a higher Killip class and/or heart rate upon admission compared to those without no-reflow (*P *< .001 and *P *< .001, respectively). Regarding laboratory and echocardiographic findings, no-reflow patients had higher white blood cell (WBC) counts, plasma glucose levels, BUN, sodium, C-reactive protein (CRP), plasma osmolarity, peak creatinine kinase myocardial band, and/or peak troponin-I as well as lower levels of LVEF and eGFR (*P *< .05 each). The frequency of the left anterior descending (LAD) artery as the IRA, total ischemic time, door-to-balloon time, proximal/ostial lesion, number of diseased arteries, the presence of chronic total occlusion (CTO), pre-TIMI flow < 3, high-grade thrombus burden, and stent length were significantly higher in patients with no-reflow (*P *< .05 each). The mean plasma osmolarity level was 300.6 ± 9.4 mOsmol/L in patients with no-reflow, while it was 292.8 ± 10.5 mOsmol/L in patients without no-reflow (*P *< .001). The incidence of cardiogenic shock as a complication of STEMI was 4.7% (n = 61) in the study. We observed that 12.3% (n = 159) of patients experienced contrast-induced acute kidney injury (CI-AKI) after pPCI. Of these, 3.7% (n = 6) of patients required hemodialysis. A total of 40 deaths occurred during the inhospital course. Notably, no-reflow patients showed a higher inhospital mortality rate than those without no-reflow [10.3% of patients (n = 29) vs. 1.1% of patients (n = 11), *P *< .001, respectively]. Patients with no-reflow had a significantly higher incidence of death compared to those without no-reflow, as observed in the Kaplan–Meier analyses [*P* (log-rank) < .001; Figure 1].

In line with the increased plasma osmolarity tertiles, the study population was categorized into 3 groups, reflecting its composition: the low tertile group (n = 431) consisted of patients with a plasma osmolarity level < 289 mOsmol/L; the intermediate tertile group (n = 432) consisted of patients with a plasma osmolarity level between 289 mOsmol/L and 298.9 mOsmol/L; and the higher tertile group (n = 431) encompassed patients with a plasma osmolarity level > 298.9 mOsmol/L. These groups are demonstrated in Table 2. Most of the aforementioned variables in Table 1 were also found to be statistically significant in patients with higher plasma osmolarity. Additionally, there was a statistically significant increase in angiographic no-reflow occurrence with higher plasma osmolarity (1.6% vs. 29.4% vs. 34.1%, *P *< .001). Furthermore, the incidences of cardiogenic shock, CI-AKI, and the inhospital mortality rate were significantly higher in patients with a higher plasma osmolarity than those with an intermediate and lower plasma osmolarity.

The impact of different variables on no-reflow was analyzed by utilizing logistic regression analyses, as displayed in Table 3. Age, DM, eGFR, smoking, a Killip class > 1, heart rate, WBC count, CRP, total ischemic time, door-to-balloon time, the LAD as the IRA, proximal/ostial lesions, TIMI flow < 3, the presence of CTO, and plasma osmolarity were found to be predictors of no-reflow by univariate analysis. In multivariate analyses, using a model adjusted for the following mentioned parameters, a Killip class > 1 (OR: 1.658; 95% CI, 1.121-2.452; *P *= .011), WBC count (OR: 1.054; 95% CI, 1.009-1.101; *P *= .018), eGFR (OR: 0.992; 95% CI, 0.986-0.999; *P* = .017), CRP (OR: 1.020; 95% CI, 1.007-1.032; *P *= .002), total ischemic time (OR: 1.003; 95% CI, 1.002-1.004; *P *< .001), pre-TIMI flow < 3 (OR: 7.872; 95% CI, 2.742-22.604; *P* < .001), proximal/ostial lesion (OR: 2.012; 95% CI, 1.432-2.827; *P *< .001), and plasma osmolarity (OR: 1.061; 95% CI, 1.045-1.076; *P *< .001) were found to be independent predictors of angiographic no-reflow.

**Figure 2.**
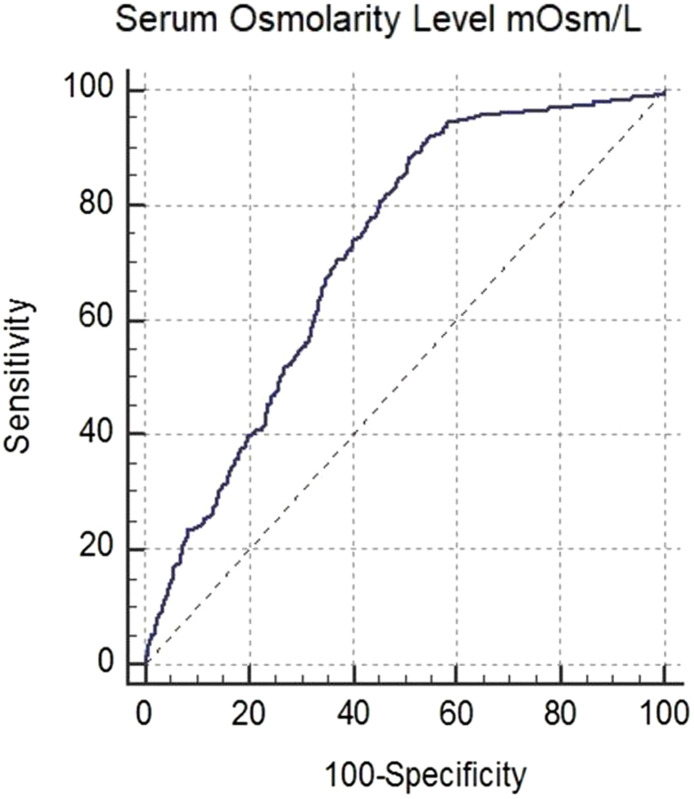
Receiver operating characteristic curve value of plasma osmolarity (mOsm/L) for no-reflow.

In the receiver operating characteristic (ROC) curve analysis, the area under the ROC curve value of the plasma osmolarity for no-reflow was 0.713 (95% CI, 0.688-0.738; *P *= 0.015; Youden’s *J* statistic: 0.3762; Figure 2). The optimum cutoff value for the plasma osmolarity determined via the ROC curve analysis was > 290.2 mOsmol/L, with a sensitivity of 91.8% and a specificity of 45.8%.

## Discussion

This is the first research to describe a significant relation between plasma osmolarity and no-reflow among STEMI patients treated with pPCI. As well an easily and simple obtained laboratory parameter, plasma osmolarity may be used for the prediction of no-reflow in patients with STEMI who have undergone pPCI.

Currently, pPCI is the recommended treatment in patients with STEMI for the restoration of normal blood flow in the IRA. However, patients may achieve epicardial coronary artery reperfusion while not achieving myocardial reperfusion after pPCI, which is referred to as no-reflow.^1^ Even though there has been a major advancement in PCI devices and techniques during the past few decades, the reported incidence of no-reflow after pPCI persists relatively frequent, and it may vary from as low as 2.3% to as high as 39.9% based on the angiographic criteria used.^2^ Similar to these previous studies, 21.7% of our study population had no-reflow. In previous studies, some clinical findings—such as the Killip class and delayed reperfusion (total ischemia time)—as well as non-cardiac laboratory parameters—such as CRP, WBC count, etc.—have been demonstrated as no-reflow’s predictors in patients with STEMI who have been managed with pPCI.^6,7,25^ Also, spesific angiographic and procedural characteristics, such as the proximal/ostial lesion as the IRA implanted stent length and TIMI flow pre-pPCI, have been reported to be independently related with no-reflow development.^5-7^ Aligning with the aforementioned studies, these variables were also found to be no-reflow’s independent predictors in our study.

Following pPCI in STEMI patients, the occurrence of no-reflow is a frequent complication; hence, its management is of critical importance in daily clinical practice. Until now, some pharmacological and mechanical strategies have been used as prophylactic treatments in order to decrease the incidence of no-reflow among STEMI patients. For example, in a prior study, it was demonstrated that patients who were treated with intracoronary adenosine had a lower incidence of no-reflow compared to those who were not treated.^26^ Kunichika et al^27^ revealed that tirofiban, which is a glycoprotein receptor inhibitor IIb/IIIa, might decrease the extent of infarct and no-reflow incidence. Silva-Orrego et al^28^ demonstrated that the use of manual thrombus aspiration before pPCI was linked with a decreased risk of no-reflow compared to the standard pPCI.

Previously, the effects of an elevated level of plasma glucose on the coronary microvasculature were investigated in some experimental studies.^29-31^ The results of these studies suggested that elevated plasma glucose, which is a main component of plasma osmolarity, enhances the aggravation of platelet-dependent thrombosis, causes vasoconstriction by affecting nitric oxide’s availability, and increases myocyte apoptosis, thereby leading to microvascular dysfunction. In common with these experimental studies’ findings, Iwakuraet al^12^ reported that acute hyperglycemia was a no-reflow’s independent predictor in 146 consecutive patients with a first acute myocardial infarction. In addition, 1 retrospective study, conducted by Ishiharaet al^13^, demonstrated that the frequency of no-reflow was significantly more common in patients with acute hyperglycemia, most likely due to microvascular dysfunction. Similarly, raised BUN levels, which is further a major component of plasma osmolarity, may lead to endothelial dysfunction, oxidative stress, and increased coagulation, particularly factor VIII activity.^32^ In a study of recent date pursued by Sensoy et al^,14^ they reported that renal dysfunction was associated with the no-reflow phenomenon in STEMI patients who underwent pPCI and manual thrombus aspiration. The investigators thought that renal impairment was associated with a hypercoagulable state, hence causing more thrombus burden, which would lead to more distal embolization during the PCI procedure.

The cardiac effects of elevated osmolarity due to hypernatremia were investigated in a previous experimental animal study.^33^ The researchers found that hypernatremia might cause decreased cardiac contractility owing to the depletion of intracellular calcium currents, most likely from sodium–calcium anti-ports. In addition, the elevated levels of osmolarity might also cause a decrease in the cardiac contractility, systemic blood pressure, and coronary blood flow. The authors speculated that the probable mechanisms behind these effects were increased intracellular viscosity, true intracellular dehydration, and embarrassed contractile elements.^33^ The observed associations between hypernatremia and decreased cardiac function were confirmed by a prospective cohort study. In this study, the authors aimed to evaluate the cardiac outcomes and mortality after a subarachnoid hemorrhage. They showed that there was a strong association between hypernatremia and LV contractile dysfunction, elevated serum levels of cardiac troponin-I, and pulmonary edema.^34^

Plasma osmolarity is composed of plasma glucose, sodium, and BUN, and it has a vital importance in maintaining the water distribution between the intracellular and extracellular compartments in the human body. Several observational studies have reported that the measurement of plasma osmolality (plasma osmolarity like) is an important assessment and laboratory tool for decision-making, especially in seriously ill patients.^35^ The predictive value of plasma osmolality and osmolarity has also been examined in patients with acute coronary syndrome, heart failure, and acute pulmonary embolism.^36-39^ These studies reported that higher plasma osmolarity at the time of admission was related to an elevated inhospital and long-term mortality rate among these patients. Yet, the suitability of plasma osmolarity for the prediction of no-reflow in STEMI patients who are managed with pPCI continues to be unknown. The current study may be the first to indicate that plasma osmolarity after multivariate analysis is independently linked to the development of no-reflow in STEMI patients. Also, we demonstrated a stepwise increase in no-reflow occurrence in line with tertiles of plasma osmolarity.

In the present research, some aspects should be considered when explaining the underlying mechanism. In the first place, an elevation of plasma osmolarity usually occurs due to the increase of its main components, such as hyperglycemia and elevation of BUN levels, both of which have distinctly been reported as risk factors in patients who developed no-reflow. Secondly, plasma osmolarity itself could cause the activation of the coagulation pathways, impair microvascular function, and aggravate platelet-dependent thrombosis, thereby resulting in more frequent no-reflow phenomena. Finally, higher plasma osmolarity might be an indicator of the severity of the underlying disease in some patients. However, since the present research was a retrospective cohort study and the underlying mechanisms remain unclear, further prospective and large-scale studies are needed to validate our findings and results.

### Limitations

Before interpreting the results of the current study, it is important to acknowledge the limitations inherent in scientific research. Despite the inclusion of a fairly large cohort with consecutive patient enrollment, the present study was conducted using a retrospective design. Given that our study focused specifically on patients with STEMI who underwent pPCI, it is crucial to acknowledge that our findings may not be applicable to all patients with acute coronary syndrome. Since STEMI types (localizations) were not recorded in our study, subgroup analysis could not be performed. Even though a multivariate analysis was performed, there is a potential existence of residual confounding from unmeasured variables, which might affect the overall outcome of the study. In addition, no-reflow was only presented by visual assessment, and more sensitive and specific methods, including cardiac magnetic resonance imaging or coronary flow reserve, were not performed concurrently. The direct measurement of plasma osmolality was not possible in our study due to the retrospective design. It is known that plasma osmolarity and osmolality are not exactly the same. Therefore, despite attentive consideration of the optimal osmolarity equation, this might have caused a minor deviation from the actual plasma osmolality values. However, the direct measurement of plasma osmolality is not routinely undertaken in most of the countries because of its limited cost-effectiveness. In our retrospective study, no-reflow treatments were not documented as they were not predetermined. For future studies involving larger patient cohorts, investigating subgroups based on no-reflow treatments is recommended. Additionally, our study had 2 major limitations: all patients received HMWH and data regarding patients’ use of oral anticoagulants before STEMI were not included. Consequently, the impact of anticoagulant therapies on outcomes could not be analyzed.

This is the first scientific study that has demonstrated a predictive value of plasma osmolarity for no-reflow development amongst STEMI patients who were managed with pPCI.

## Figures and Tables

**Table 1.** The Baseline Demographic Characteristics and Laboratory and Angiographic Findings of All Patients

	All Patients, n = 1294	NR (−), n = 1013	NR (+), n = 281	*P*
Age, years	56 ± 12	55 ± 12	59 ± 13	<.001
Female gender, n (%)	241 (18.6)	183 (18.1)	58 (20.6)	.327
History	3			
Diabetes mellitus, n (%)	297 (23.0)	203 (20.0)	94 (33.5)	<.001
Hypertension, n (%)	514 (39.7)	393 (38.8)	121 (43.1)	.196
Smoking, n (%)	710 (54.9)	586 (57.8)	124 (44.1)	<.001
On admission	3			
Killip class >1, n (%)	201 (15.5)	111.0 (11.0)	90.0 (32.0)	<.001
Systolic blood pressure, mm Hg	131 ± 31	131 ± 28	130 ± 40	.461
Heart rate, bpm	77 ± 16	76 ± 14	80 ± 19	<.001
Laboratory findings	3			
White blood cell count, 10^3^/uL	12.3 ± 3.7	11.9 ± 3.3	13.9 ± 4.4	<.001
Hemoglobin, g/dL	13.8 ± 1.7	13.8 ± 1.7	13.8 ± 1.8	.976
Blood glucose, g/dL	146.6 ± 71.1	139.0 ± 64.6	173.8 ± 85.7	<.001
Blood urea nitrogen level, mg/dL	15.8 ± 5.7	15.2 ± 4.9	18.2 ± 7.7	<.001
Sodium, mmol/L	139 ± 4	139 ± 4	141 ± 4	<.001
C-reactive protein, mg/dL	8.0 ± 3.4	7.5 ± 3.2	12.0 ± 4.7	<.001
eGFR, mL/min	89.8 ± 24.8	92.2 ± 23.5	81.2 ± 27.4	<.001
Peak troponin I, ng/mL	76.2 (35-159)	60.9 (27-119.9)	178 (92.9-302.8)	<.001
Peak CK-MB, U/L	167 ± 93	136 ± 83	342 ± 208	<.001
Plasma osmolarity, mOsmol/L	294.5 ± 10.7	292.8 ± 10.5	300.6 ± 9.4	<.001
Angiographic outcomes	3	3	3	3
IRA, n (%)	3	3	3	3
LAD	642 (49.6)	481 (47.5)	161 (57.3)	.001
Cx	179 (13.8)	138 (13.6)	41 (14.6)	
RCA	452 (34.9)	378 (37.3)	74 (26.3)	
Others	21 (1.6)	16 (1.6)	5.0 (1.8)	
Left ventricular EF, %	47 ± 8.5	48.8 ± 7.8	40.6 ± 8.0	<.001
Door-to-balloon time, minutes	31 ± 9	30 ± 9	32 ± 7	<.001
Total ischemic time, minutes	170 ± 110	150 ± 102	222 ± 145	<.001
Proximal or ostial lesion, n (%)	675 (52.2)	463 (45.7)	212 (75.4)	<.001
LMCA, n (%)	6 (0.5)	3 (0.3)	3 (1.1)	.092
Number of diseased vessels, n (%)	3	3	3	3
1	820 (63.4)	654 (64.6)	166 (59.1)	.043
2	368 (28.4)	286 (28.2)	82 (29.2)	
3	106 (8.2)	73 (7.2)	33 (11.7)	
Presence of CTO, n (%)	52 (4.0)	28 (2.8)	24 (8.5)	<.001
Pre-TIMI flow < 3, n (%)	1152 (89.0)	875 (86.4)	277 (98.6)	<.001
High-grade thrombus, n (%)	841 (65.0)	614 (60.6)	227 (80.8)	<.001
Stent diameter, mm	3.11 ± 0.36	3.10 ± 0.33	3.16 ± 0.43	.118
Stent length, mm	21.5 ± 8.7	20.7 ± 8.1	24.5 ± 19.4	<.001
Inhospital outcomes	3	3	3	3
Cardiogenic shock, n (%)	61 (4.7)	21 (2.1)	40 (14.2)	<.001
CI-AKI, n (%)	159 (12.3)	100 (9.9)	59 (21.2)	<.001
Inhospital mortality, n (%)	40 (3.1)	11 (1.1)	29 (10.3)	<.001
Length of hospital stay, days	4 ± 3	4 ± 3	5 ± 4	<.001

CI-AKI, contrast-induced acute kidney injury; CK-MB, creatinine kinase myocardial band; CTO, chronic total occlusion; Cx, circumflex; eGFR, estimated glomerular filtration rate; EF, ejection fraction; IRA, infarct-related artery; LAD, left anterior descending; LMCA, left main coronary artery; NR, no-reflow; RCA, right coronary artery; TIMI, thrombolysis in myocardial infarction.

**Table 2.** The Baseline Demographic Characteristics and Laboratory, and Angiographic Findings in Accordance of Plasma Osmolarity (mOsm/L) Tertiles with *P*-Value

	289 < Plasma Osmolarity, n = 431	289-298.9 Plasma Osmolarity, n = 432	>298.9 Plasma Osmolarity, n = 431	*P*
Age, years	55 ± 12	56 ± 12	57 ± 12	.065
Female gender, n (%)	77 (17.9)	71 (16.4)	93 (21.6)	.135
History	3			
Diabetes mellitus, n (%)	48 (11.1)	76 (17.6)	173 (40.1)	<.001
Hypertension, n (%)	167 (38.7)	153 (35.4)	194 (45)	.014
Smoking, n (%)	244 (56.6)	241 (55.8)	225 (52.2)	.385
On admission	3			
Killip class >1, n (%)	52 (12.1)	60 (13.9)	89.0 (20.6)	.001
Systolic blood pressure, mm Hg	131 ± 27	130 ± 29	132 ± 36	.608
Heart rate, bpm	77 ± 15	77 ± 15	78 ± 17	.279
Laboratory findings	3			
White blood cell count,10^3^/uL	11.7 ± 3.3	12.4 ± 3.6	12.8 ± 4	<.001
Hemoglobin, g/dL	13.9 ± 1.6	13.9 ± 1.8	13.7 ± 1.8	.483
Blood glucose, g/dL	116.2 ± 29.2	133.6 ± 47.0	190 ± 95.7	<.001
Blood urea nitrogen level, mg/dL	14.9 ± 4.9	15.6 ± 5.4	17 ± 6.6	<.001
Sodium, mmol/L	135 ± 2	140 ± 2	143 ± 3	<.001
C-reactive protein, mg/dL	7.5 (2.8-14.3)	7.75 (3.2-14.4)	9.2 (4.5-16.7)	.015
eGFR, mL/m	91.4 ± 22.7	90.9 ± 25.5	87.1 ± 26	.085
Peak troponin I, ng/mL	56.3 (25-131)	78 (35.9-165.3)	90 (45-189)	<.001
Peak CK-MB, U/L	134 (78-231)	178 (98-308)	189 (113-325)	<.001
Plasma osmolarity, mOsmol/L	282.9 ± 3.8	293.8 ± 2.7	306.6 ± 6.6	<.001
Angiographic outcomes	3	3	3	3
IRA, n (%)	3	3	3	3
LAD	194 (45)	226 (52.3)	222 (51.5)	.146
Cx	72 (16.7)	60 (13.9)	47 (10.9)	
RCA	157 (36.4)	139 (32.2)	156 (36.2)	
Others	8 (1.9)	7 (1.6)	6.0 (1.4)	
Left ventricular EF, %	49 ± 7.8	46.6 ± 8.2	45.3 ± 9.1	<.001
Door to balloon time, min	31±11	30±6	32±7	.019
Total ischemic time, min	148 (100-242)	173 (106-255)	180 (118-262)	.015
Proximal or ostial lesion, n (%)	185 (42.9)	240 (55.6)	250 (58)	<.001
LMCA, n (%)	2 (0.5)	3 (0.7)	1 (0.2)	.610
Number of diseased vessels, n (%)	3	3	3	3
1	285 (66.1)	275 (63.7)	260 (60.3)	.160
2	117 (27.1)	122 (28.2)	129 (29.9)	
3	29 (6.7)	35 (8.1)	42 (9.7)	
Presence of CTO, n (%)	10 (2.3)	17 (3.9)	25 (5.8)	.030
Pre-TIMI flow <3, n (%)	378 (87.7)	380 (88)	394 (91.4)	.150
High-grade thrombus, n (%)	268 (62.2)	285 (66)	288 (66.8)	.320
Stent diameter, mm	3.11 ± 0.34	3.10 ± 0.35	3.12 ± 0.37	.650
Stent length, mm	20.5 ± 7.5	21.2 ± 8.2	22.8 ± 10.2	.010
No-reflow, n (%)	7 (1.6)	127 (29.4)	147 (34.1)	<.001
Inhospital outcomes	3	3	3	3
Cardiogenic shock, n (%)	7 (1.6)	21 (4.9)	33 (7.7)	<.001
CI-AKI, n (%)	38 (8.8)	51 (11.9)	70 (16.4)	<.001
Inhospital mortality, n (%)	2 (0.5)	14 (3.2)	24 (5.6)	<.001
Length of hospital stay, days	4±3	4±3	4±3	<.001

CI-AKI, contrast-induced acute kidney injury; CK-MB, creatinine kinase myocardial band; CTO, chronic total occlusion; Cx, circumflex; EF, ejection fraction; eGFR, estimated glomerular filtration rate; IRA, infarct-related artery; LAD, left anterior descending; LMCA, left main coronary artery; NR, no-reflow; RCA, right coronary artery; TIMI, thrombolysis in myocardial infarction.

**Table 3.** Univariate Analysis and Multivariate Model for No-Reflow

	Univariate Analysis		Multivariate Analysis	
	*P*	OR (95% CI)	*P*	OR (95% CI)
Age	<.001	1.025 (1.014-1037)	—	—
Diabetes mellitus	<.001	2.006 (1.498-2.685)	—	—
Smoking	<.001	0.576 (0.441-0.751)	—	—
Killip class >1	<.001	3.829 (2.784-5.267)	0.011	1.658 (1.121-2.452)
Heart rate	<.001	1.016 (1.007-1.025)	—	—
White blood cell count	<.001	1.159 (1.118-1.202)	0.018	1.054 (1.009-1.101)
eGFR	<.001	0.981 (0.976-0.987)	0.017	0.992 (0.986-0.999)
C-reactive protein	<.001	1.047 (1.035-1.059)	0.002	1.020 (1.007-1.032)
Door-to-balloon time	.004	1.026 (1.008-1.045)	—	—
Total ischemic time	<.001	1.004 (1.003-1.005)	<0.001	1.003 (1.002-1.004)
LAD as the IRA	.001	0.791 (0.685-0.914)	—	—
Pre-TIMI flow<3	<.001	10.92 (4.005-29.78)	<0.001	7.872 (2.742-22.604)
Proximal or ostial lesion	<.001	3.650 (2.708-4.919)	<0.001	2.012 (1.432-2.827)
Presence of CTO	<.001	3.285 (1.872-5.764)	—	—
Plasma osmolarity	<.001	1.090 (1.073-1.108)	<0.001	1.061 (1.045-1.076)

CTO, chronic total occlusion; eGFR, estimated glomerular infiltration rate; IRA, infarct-related artery; LAD, left anterior descending artery; OR, odds ratio; TIMI, thrombolysis in myocardial infarction.
